# Comparing Aging and Fitness Effects on Brain Anatomy

**DOI:** 10.3389/fnhum.2016.00286

**Published:** 2016-06-28

**Authors:** Mark A. Fletcher, Kathy A. Low, Rachel Boyd, Benjamin Zimmerman, Brian A. Gordon, Chin H. Tan, Nils Schneider-Garces, Bradley P. Sutton, Gabriele Gratton, Monica Fabiani

**Affiliations:** ^1^Beckman Institute, University of Illinois, UrbanaIL, USA; ^2^Neuroscience Program, University of Illinois, UrbanaIllinois, USA; ^3^Department of Psychology, University of Illinois, UrbanaIL, USA; ^4^Department of Radiology, Washington University in St. Louis, Saint LouisMO, USA; ^5^Department of Bioengineering, University of Illinois at Urbana–Champaign, UrbanaIL, USA

**Keywords:** aging, cardiorespiratory fitness, exercise, brain anatomy, FreeSurfer

## Abstract

Recent studies suggest that cardiorespiratory fitness (CRF) mitigates the brain’s atrophy typically associated with aging, via a variety of beneficial mechanisms. One could argue that if CRF is generally counteracting the negative effects of aging, the same regions that display the greatest age-related volumetric loss should also show the largest beneficial effects of fitness. To test this hypothesis we examined structural MRI data from 54 healthy older adults (ages 55–87), to determine the overlap, across brain regions, of the profiles of age and fitness effects. Results showed that lower fitness and older age are associated with atrophy in several brain regions, replicating past studies. However, when the profiles of age and fitness effects were compared using a number of statistical approaches, the effects were not entirely overlapping. Interestingly, some of the regions that were most influenced by age were among those not influenced by fitness. Presumably, the age-related atrophy occurring in these regions is due to factors that are more impervious to the beneficial effects of fitness. Possible mechanisms supporting regional heterogeneity may include differential involvement in motor function, the presence of adult neurogenesis, and differential sensitivity to cerebrovascular, neurotrophic and metabolic factors.

## Introduction

The brains of seemingly healthy individuals undergo various degrees of cortical and subcortical atrophy with aging (Raz et al., 2005, 2010; Gordon et al., 2008). The exact mechanisms underlying these changes are not completely known, although vascular (Brown and Thore, 2011; Davenport et al., 2012; Lähteenvuo and Rosenzweig, 2012; Fabiani et al., 2014), genetic (McGue and Johnson, 2008; Papenberg et al., 2015), oxidative stress (Muller et al., 2007; Dai et al., 2014; Thorin and Thorin-Trescases, 2014) and hormonal (Morrison and Baxter, 2012) factors may all contribute significantly. These anatomical changes are considered to have important functional consequences, and to influence some of the cognitive decline that accompanies aging (e.g., Salthouse, 2011; Fabiani, 2012; Nyberg et al., 2012).

In an attempt to delay or possibly even reverse some of these structural changes, investigators have focused on lifestyle factors as potential mitigators of the effects of aging. One such factor that has received particular attention is CRF. Higher CRF is correlated with a number of systemic benefits, including lower blood pressure (Blair et al., 1996; Barlow et al., 2006), decreased stress (Jackson and Dishman, 2006), and better lipid profiles (Dunn et al., 1999; Park et al., 2015). Extensive research has also demonstrated that higher CRF is correlated with improved brain tissue preservation and higher performance in neuropsychological tests (e.g., Colcombe and Kramer, 2003; Gordon et al., 2008; Voss et al., 2011, 2013; Verstynen et al., 2012; Weinstein et al., 2012; McAuley et al., 2013). It has also been shown that CRF mediates age-related changes in some structural (e.g., frontal cortex volume, Weinstein et al., 2012; caudate nucleus, Verstynen et al., 2012) and functional measures (e.g., cerebral blood flow; Zimmerman et al., 2014), and that these changes are relevant for cognitive function (Verstynen et al., 2012; Weinstein et al., 2012). Beneficial effects of CRF on the preservation of regional brain volumes have been reported for a number of brain regions in cross-sectional comparisons of age and fitness groups (Gordon et al., 2008; Bugg and Head, 2011; Verstynen et al., 2012), as well as in response to fitness intervention (Colcombe et al., 2003, 2006; Erickson et al., 2011; Voss et al., 2011). Most of the research conducted thus far, however, has not examined the regional specificity of CRF effects on brain anatomy in a systematic manner, but has instead focused on specific brain areas (such as the hippocampus and prefrontal cortex) that are known to be affected by aging (e.g., Bugg and Head, 2011; Erickson et al., 2011; McAuley et al., 2011).

In this study, we are proposing a strategy to investigate the extent to which the effects of CRF on volumetric brain measures are generalized or regional, and their overlap with the effects of aging. In other words, are the profiles of the effects of CRF and of aging similar throughout the brain or does CRF preferentially influence only some of the brain regions that are affected by aging and not others? To address these questions we report a direct comparison of the profiles of the effects of aging and fitness on volumetric measures obtained throughout the brain (including both cortical and subcortical structures) in older adults between the ages of 55 and 87.

*In vivo* volumetric measurement of brain areas based on structural magnetic resonance imaging (sMRI) allows for researchers to study the influence of demographic and lifestyle factors on different patterns of volumetric decline in aging. Further, measurements of brain structures throughout the whole brain are critical to provide the comprehensive data required for a profile analysis. Although manual tracing remains the gold standard for *in vivo* anatomical studies based on structural MR images (Raz et al., 2003; Kennedy et al., 2009), a number of semi-automated methods have become available in the last two decades. These methods, allowing for the parallel measurement of multiple structures, permit a more complete examination of brain anatomy in large groups of individuals without requiring extensive training. One of the first automatic methods used for investigating the effects of CRF on brain anatomy was voxel-based morphometry (VBM; Ashburner and Friston, 2000), which allows for probabilistic brain mapping across populations. VBM studies of the effects of aging on brain anatomy have demonstrated significant gray and white matter loss in a wide range of areas, including most of the frontal and parietal cortex, as well as most subcortical gray matter regions (e.g., Good et al., 2002; Gordon et al., 2008). VBM studies have also examined the effects of CRF. For example, in a cross-sectional study including younger (ages 20–28) and older adults (ages 65–81) Gordon et al. (2008) showed that higher CRF was associated with volumetric preservation in the inferior frontal, anterior parietal, and medial temporal regions, even when education, age and gender were controlled for. One of the first fitness intervention studies to use VBM (Colcombe et al., 2006) demonstrated that a 6-month aerobic fitness intervention in low-fit older adults (ages 60–79) led to significant volumetric increases in the dorsal anterior cingulate, supplementary motor area, middle frontal gyrus, left superior temporal lobe, and anterior third of the corpus callosum (CC). Other VBM studies examining subcortical regions have shown that higher CRF is associated with larger hippocampal volumes, and that exercise interventions may even lead to an increase in hippocampal size (Erickson et al., 2011; McAuley et al., 2011).

Although VBM can provide a useful estimate of anatomical effects, it also has several limitations, including the fact that coregistration of different structures across individuals relies on group templates, which may introduce biases in the comparison process (Bookstein, 2001; Salmond et al., 2002; Davatzikos, 2004; Ridgway et al., 2008; Kennedy et al., 2009; Whitwell, 2009). FreeSurfer © provides an alternative approach: It offers a way of coregistering anatomical structures across individuals using a semi-automatic parcellation of the cortex based on surface and border features, and the possibility of separating cortical volume from cortical thickness (Dale and Sereno, 1993; Dale et al., 1999; Fischl et al., 2001). Using FreeSurfer, Bugg and Head (2011) examined the correlation between the amount of running, jogging, and walking (as indexed by a questionnaire looking at the previous 10 years) and loss in a few regions of interest (ROIs) in older adults (ages 55–79). Although only some regions were examined, they showed that exercise mitigated age-related atrophy in the medial temporal lobe (MTL), but failed to show an interaction between age and exercise in other ROIs (including areas of the prefrontal cortex and parietal lobes). In the current study, we used FreeSurfer to derive volumetric brain measurements in a sample of middle–aged and older adults. We also used multiple statistical methodologies in an attempt to provide a more comprehensive description of the effects of fitness and age across brain regions to evaluate their profile overlap. We believe that an improved understanding of the overlap between these variables will help design more effective intervention programs (see for example Bherer et al., 2013; Voss et al., 2013).

## Materials and Methods

### Participants

Fifty-six healthy adults (29 females, age range = 55–87) from the Champaign-Urbana community were recruited through local advertisements, mass emailing, and flyers. The study was approved by the University of Illinois Institutional Review Board, and all participants signed documents of informed consent in accordance with the Declaration of Helsinki. All participants were right-handed and fluent English speakers. Information about the participants’ demographics is reported in **Table 1**.

**Table 1:** Basic demographic, neuropsychological, and physiological characteristics.

	All (*N* = 54)		Low-fit (*N* = 27)		High-fit (*N* = 27)		*t*-test (2-tailed)	
Females: Males	29:25		15:12		14:13			
	Mean	*SD*	Mean	*SD*	Mean	*SD*	*p*	
Age (years)	69.48	8.46	70.20	8.51	68.75	8.51	0.533	
eCRF (MET)	6.58	2.47	5.28	1.97	7.88	2.25	0.000	^∗∗^
Est. VO_2max_ (ml/min/Kg)	23.03	8.64	18.47	6.88	27.58	7.86	0.000	^∗∗^
Education (years)	16.73	2.96	15.63	3.03	17.83	2.48	0.005	^∗∗^
Weight (Kg)	79.05	16.88	84.28	15.69	73.82	16.66	0.021	^∗^
BMI (Kg/m^2^)	27.46	5.27	30.06	4.59	24.87	4.64	0.000	^∗∗^
BDI score	2.91	3.34	3.63	3.64	2.19	2.91	0.114	
mMMS score	55.28	1.42	55.26	1.35	55.30	1.51	0.925	
Heart rate (bpm)	72.90	10.62	76.96	10.91	68.83	8.74	0.004	^∗∗^
Systolic (mmHg)	136.90	15.66	139.33	16.59	134.48	14.58	0.258	
Diastolic (mmHg)	79.28	8.16	79.84	8.95	78.72	7.41	0.618	
Pulse pressure (mmHg)	57.63	14.01	59.49	14.25	55.76	13.77	0.332	

*eCRF, Estimated Cardiorespiratory Fitness; MET, Metabolic Equivalent; Estimated VO_2max_, milliliters of oxygen per minute per kilogram of body weight; Kg, Kilograms; BMI, Body Mass Index – weight in Kg over height in meters squared (m^2^); bpm, beats per minute; BDI, Beck’s Depression Inventory; mMMS, Modified Mini–Mental Status Examination; Systolic and diastolic blood pressure: mmHg (millimeters of mercury). ^∗^*p* < 0.05, ^∗∗^*p* < 0.01.*

Phone interviews were conducted to screen individuals for inclusion/exclusion. Exclusion criteria included a history of drug abuse, major psychiatric or neurological disease, or other serious chronic medical conditions. Those who passed the phone screening were invited into the lab and were given two additional screening measures, the Beck’s Depression Inventory (Beck and Steer, 1987) and the modified Mini–Mental Status Exam (Mayeux et al., 1981). Individuals scoring above 14 on the BDI or 51 or less on the mMMSE were excluded from the study. Two male participants were excluded from analysis due to incomplete fitness estimates or missing neuropsychological data. Our final sample consisted of 54 participants.

The anatomical MRIs of 8 college students (age range = 19–22, Mean Age: = 21.53, *SD* = 0.99, five Females) were also used for head-size normalization but were excluded from all other analyses (see below for rationale). These younger participants reported themselves to be healthy, right-handed, fluent in English and also underwent a screening similar to the one described above.

#### Fitness Estimation

Estimated cardiorespiratory fitness (eCRF) scores were obtained for each participant using the regression model proposed by Jurca et al. (2005). The eCRF measures have been validated with a large sample of older adults (Mailey et al., 2010; McAuley et al., 2011). They are highly correlated (≈0.7) with VO_2max_, which is obtained through a graded exercise protocol and is considered the gold standard for assessing CRF. Specifically, Mailey et al. (2010) found that within a population of older adults, eCRF was significantly correlated with metabolic equivalents (METs) (*r* = 0.66) and with estimates based on submaximal field testing (*r* = 0.67). Crucially, eCRF allowed us to include participants that may be at risk during standard VO_2max_ testing.

The regression model used for eCRF estimation is based on weighted data including gender, age, body mass index (BMI), resting heart rate, and a physical activity score. Height and weight were measured to calculate the BMI. Resting heart rate was recorded on three separate days, after participants had been sitting for a minimum of 5 min, and these measurements were then averaged. The activity score was derived from the Physical Activity Scale for the Elderly (PASE, Washburn et al., 1993). The PASE contains specific questions to determine the type and frequency (minutes per session and number of sessions per week) of exercise activities a participant does on an average week. Specifically, the total minutes per week of exercises requiring low exertion (i.e., activities that produce only a slight increase in heart rate) and those classified as aerobic exercise (jogging, swimming, cycling etc.) were quantified. These totals were used to categorize each participant into one of the five self-reported activity scores defined by Jurca et al. (2005), and then combined with the other weighted data to obtain an eCRF value for each participant. The eCRF score is expressed in METs, which are defined as the amount of oxygen consumed while sitting at rest (see Jetté et al., 1990). To estimate VO_2max_ from the METs values, the eCRF score needs to be multiplied by 3.5 (Masley, 2009). To facilitate cross-referencing with studies measuring VO_2max_, **Table 1** also reports the VO_2max_ values estimated from eCRF. The means of the fitness estimates for each group, when compared to existing norms, indicate that they correspond to meaningful differences, with the low-fitness group classified as “poor prognosis in coronary patients; highly deconditioned individuals” (Jurca et al., 2005; see also McArdle et al., 2006).

#### Sample Stratification and Analytic Strategies

The purpose of the present study is to *independently* assess the effects of age and fitness on brain anatomy. A critical step is to “orthogonalize” these two variables, which are typically highly correlated. To do so, participants were sorted into high- and low-fit groups using the following strategy. To control for the effects of age and gender on fitness, group membership was determined by first separating participants by age, split by the average age of the entire participant pool (mean = 69.56 years). Within each age group participants were then divided by gender and then split by eCRF scores using a median split for each age by gender group. In groups with uneven numbers of members, median participants were designated as high- or low-fit based on whether their eCRF scores were above or below the average eCRF scores of their peer group. Next, participants were combined into high-fit and low-fit categories collapsing across all other categorizations. Fitness designation was done in this way to account for the heavy weighting that gender and age receive in the CRF estimate, and to control for the gender bias known to exist within VO_2max_ scores (Jurca et al., 2005). Thus, each individual was classified as high- or low-fit based on a comparison with their similarly aged peers within the same gender group. As a result of this grouping strategy (see **Table 1**) the low-fit and high-fit groups had an almost equivalent number of males and females (14F, 13M), statistically similar average age (70.30 versus 68.75, *t* = 0.63, *p* > 0.10), but significantly different average eCRF scores (5.28 versus 7.88, *t* = 4.53, *p* < 0.01), thus effectively stratifying the sample and eliminating the inherent correlation between age and fitness.

### Collection and Processing of Structural MRI Data

High-resolution T1-weighted images were acquired with a 3T Siemens Trio full body scanner using a 3D MPRAGE protocol. MPRAGE pulse parameters were: flip angle = 9, TR = 1900 ms, TE = 2.32 ms, and inversion time = 900 ms. Slices were acquired in the sagittal plane (192 slices, 0.9 mm slice thickness, voxel size 0.9 mm × 0.9 mm × 0.9 mm) with matrix dimensions of 192 × 256 × 256 (in-plane interpolated at acquisition to 192 × 512 × 512) and field of view of 172.8 mm × 230 mm × 230 mm. These parameters allowed for a clear delineation of gray–white matter boundaries upon visual inspection. Structural MRI images were processed with FreeSurfer © 5.0 (for technical details see Dale and Sereno, 1993; Dale et al., 1999; Fischl et al., 1999a,b, 2001, 2002, 2004; Fischl and Dale, 2000; Segonne et al., 2004; Desikan et al., 2006; Han et al., 2006; Jovicich et al., 2006). The FreeSurfer output was thoroughly inspected for errors through extensive visual screening performed by three trained individuals, and corrected according to the methods recommended on the FreeSurfer web site^1^. Finally, estimates of cortical and subcortical volumes were obtained, using an automated probabilistic labeling procedure based on the Desikan–Killiany anatomical atlas (Desikan et al., 2006).

Subcortical and cortical regions were then normalized by intracranial volume (see equation below) to account for volumetric differences in head size (Jack et al., 1989; Buckner et al., 2004). The anatomical data from eight college-aged students were also included in the normalization process in order to frame the results obtained from our 55–87 years-old sample into a broader age-related perspective. In other words, we included the young adults as a reference point, so that the normalized values can be more easily compared to those obtained from samples that, unlike this one, do include younger adults. Mathematically normalization consists of a scaling factor that operates equally for all subjects and groups and therefore does not introduce any bias. Every volume for each participant was corrected for head size with the following formula proposed by Buckner et al. (2004):

$${\text{V}}_{\text{adj}}{\text{=V}}_{\text{nat}}\text{-b}({\text{eTIV}}_{\text{fs}}\text{-}{\bar{\text{eTIV}}}_{\text{fs}})$$

where V_adj_ is the covariance-corrected (adjusted) volume, V_nat_ is the original volume calculated by FreeSurfer in native space, b is the slope of the regression of V_nat_ over eTIV_fs_, which is the participant’s eTIV produced by FreeSurfer, and 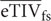 is the average eTIV of the group. Although several other normalization methods have been proposed, this particular one has been widely utilized throughout the field.

#### Statistical Analyses

The processed and corrected data were analyzed using two approaches: (a) a standard analysis evaluating all the cortical and subcortical ROIs included in the Desikan–Killiany atlas (Desikan et al., 2006); and (b) groupings of different cortical and subcortical regions showing similar effects based on a principal component analysis/VARIMAX decomposition (see Alexander-Bloch et al., 2013), in order to reduce the problem of multiple comparisons present in (a). The choice of an empirical, data-driven (rather than a theory-driven) approach for grouping the different regions was motivated by the following logic: (a) At present, the factors leading some regions more than others to be affected by aging or fitness are not yet known, and this study is, in fact, one of the first addressing this issue; and (b) the factor-analytic approach employed here allows for the data to reveal emergent groupings, which may be useful to drive future research aimed at understanding the mechanisms underlying regional variations in sensitivity to age and fitness. For all these analyses, left and right hemispheres were combined, as we had no specific hemispheric-based hypotheses.

To dissociate the effects of age and fitness we used a data-driven stratification approach, in which participants were given an “age-score” equal to the deviation of their age from the overall mean age (yielding two groups of participants above and below the overall mean age, respectively), and a “fitness-score” with a value of +1 for “high-fit-for-their-age” individuals (defined as above) and -1 for “low-fit-for-their-age individuals. This approach allowed also for the examination of age × fitness interactions, testing whether age effects were similar for high- and low-fit individuals (i.e., whether fitness moderates the effect of age), a pattern of results that is not typically examined by studies using a more traditional multiple regression approach. Pearson’s correlation coefficients (*r*) were calculated to determine the associations of age, fitness and the age × fitness interaction with neuroanatomical measurements. Also, a multiple regression analysis was performed to determine the unique variance explained by these three variables. For this analysis, given the directional nature of the hypotheses (i.e., younger and more fit individuals should have larger brain volumes than older and less fit individuals), we used unidirectional tests.

The main purpose of this study is to compare the profiles of anatomical effects associated with aging and fitness. For this analysis we selected data from the ROI approach. For each of the 48 Desikan–Killiany areas, the effect sizes associated with age and fitness were calculated using Cohen’s *d*. The similarity of the effect sizes of age and fitness across anatomical areas was estimated using a Pearson’s correlation coefficient. A consistency-based two-way mixed intra-class correlation coefficient was also calculated to estimate the consistency of the profiles of regional volumetric changes with age and fitness.

## Results

### Age and Fitness Effects: Demographics

Age, fitness, and various physiological and neuropsychological measures are reported in **Table 1**. Several variables demonstrated negative correlations with age including BMI [*r*(52) = -0.48, *p* < 0.01] and diastolic pressure [*r*(52) = -0.39, *p* < 0.01]. Pulse pressure (the difference between systolic and diastolic pressure, indexing arterial stiffness) was positively correlated with age [*r*(52) = 0.36, *p* < 0.01].

### Regional Analyses of Age and Fitness Effects

To determine that the lack of correlation or consistency between the effect of age and fitness on brain volume is not merely due to lack of power, it is important to show that regional volumetric measures are sensitive to each of these two independent variables. To this end, we performed a set of multiple regressions investigating the effects of age, fitness, and their interaction on each individual area. A problem with this analysis is that it is based on a large number of comparisons, which are only partially independent of each other. Therefore, we also performed a second analysis in which we first grouped the different areas using a factor analysis (i.e., PCA followed by Varimax rotation).

#### Region by Region Analysis

**FIGURE 1:**
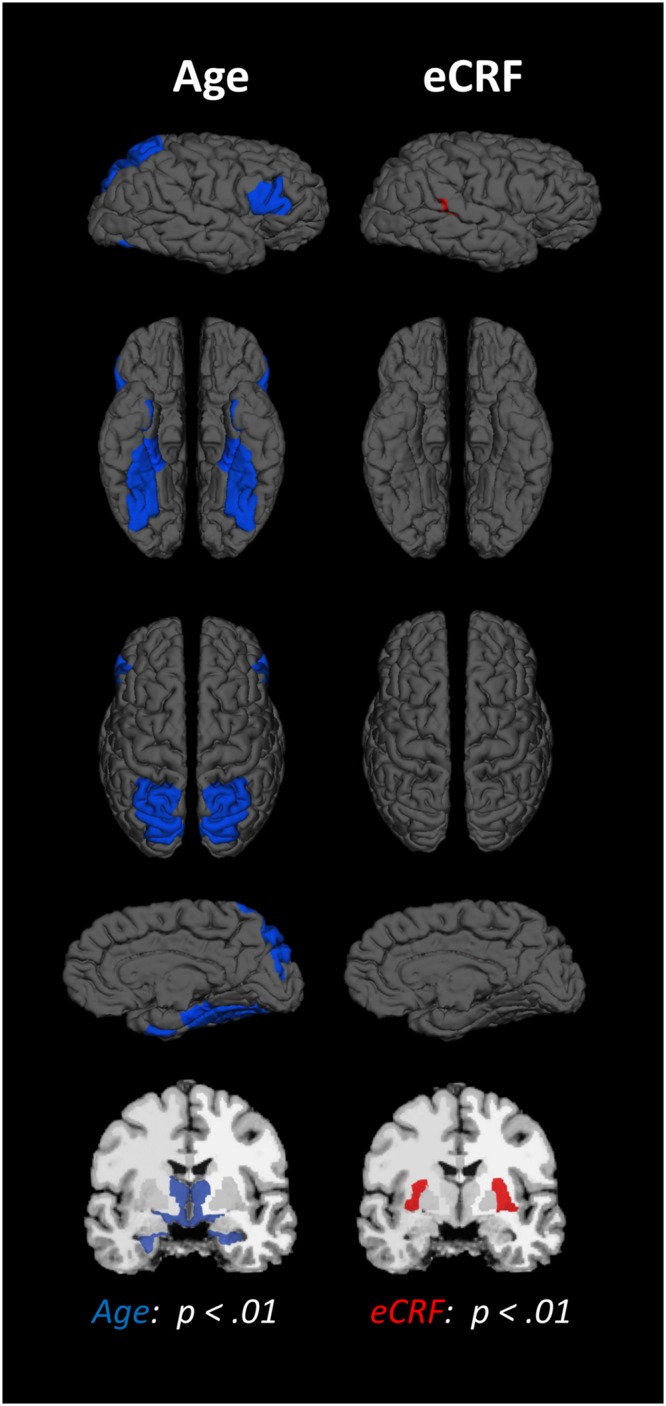
**Schematic representation of the results of the regression analysis for all of the ROIs.** Colored areas indicate ROIs where age (blue) or eCRF (red) show strong partial correlations (*p* < 0.01) with the volume of the corresponding region. Although left and right volumes were not examined separately, for illustration purposes both hemispheres are included in the figure. Note that no ROI was significantly affected by both variables in this analysis.

The BERT template provided by FreeSurfer was colored (**Figure 1**) to provide a visual summary of cortical and subcortical regions of the Desikin–Killiany atlas that were most strongly (*p* < 0.01) associated with age and fitness. The correlations between age, fitness and the volumes of cortical ROIs are found in **Table 2**. Within the frontal lobe, only the pars opercularis, pars triangularis and precentral regions were found to be significantly negatively associated with age. However, the superior frontal and precentral regions were positively associated with fitness. In the temporal lobe, most of the regions were negatively associated with age. Furthermore, the superior temporal sulcus was positively associated with fitness, whereas superior temporal and middle temporal areas showed a similar, but not significant, trend (*p* < 0.10). The only region showing a modest interaction effect between age and fitness was the fusiform gyrus, with larger volumes in high-fit younger adults, but smaller volumes in high-fit older adults. All the sub-regions of the parietal lobe were negatively associated with age, but none were positively associated with eCRF. The insula was not associated with either age or eCRF. The posterior cingulate was associated with both age and eCRF, whereas the caudal anterior cingulate was associated only with eCRF.

**Table 2:** Raw and partial correlations between anatomical ROIs and age, eCRF, and eCRF by age interaction (centered age multiplied by dichotomized fitness scores).

	Raw Correlations						Partial Correlations					
	Age (r)		eCRF (r)		Age × eCRF (r)		Age (β)		eCRF (β)		Age × eCRF (β)	
**Frontal Lobe**												
Superior Frontal	-0.18		0.23	+	0.13		-0.17		0.22		0.13	
Rostral Middle Frontal	-0.09		-0.09		-0.05		-0.10		-0.09		-0.05	
Caudal Middle Frontal	-0.22		-0.01		0.21		-0.23		-0.03		0.21	
Pars opercularis	-0.34	^∗^	0.11		0.18		-0.34	^∗^	0.08		0.20	
Pars triangularis	-0.42	^∗∗^	0.12		0.00		-0.42	^∗∗^	0.09		0.00	
Pars orbitalis	-0.18		0.09		0.13		-0.18		0.08		0.13	
Lateral Orbitofrontal	-0.13		-0.07		0.06		-0.14		-0.09		0.06	
Medial Orbitofrontal	-0.12		0.19		0.14		-0.11		0.18		0.14	
Frontal Pole	0.10		-0.07		-0.05		0.09		-0.06		-0.05	
Precentral	-0.32	^∗^	0.28	^∗^	0.10		-0.31	^∗^	0.26	+	0.11	
Paracentral	-0.20		0.16		-0.13		-0.19		0.15		-0.14	
**Temporal Lobe**												
Superior Temporal	-0.33	^∗^	0.22		0.21		-0.33	^∗^	0.21		0.23	
Middle Temporal	-0.23	+	0.22		0.12		-0.22		0.21		0.13	
Inferior Temporal	-0.14		-0.12		0.03		-0.15		-0.13		0.03	
Banks of the STS	-0.31	^∗^	0.41	^∗∗^	0.01		-0.30	^∗^	0.41	^∗∗^	0.01	
Fusiform	-0.36	^∗∗^	0.09		-0.24	+	-0.37	^∗∗^	0.06		-0.25	+
Transverse Temporal	-0.13		0.22		0.09		-0.12		0.21		0.09	
Entorhinal	-0.25	+	0.19		-0.01		-0.24	+	0.18		-0.02	
Temporal Pole	-0.35	^∗∗^	0.06		0.20		-0.35	^∗^	0.03		0.21	
Parahippocampal	-0.48	^∗∗^	0.07		0.08		-0.47	^∗∗^	0.03		0.09	
**Parietal Lobe**												
Superior Parietal	-0.45	^∗∗^	0.02		0.02		-0.45	^∗∗^	-0.02		0.02	
Inferior Parietal	-0.33	^∗^	0.15		0.06		-0.32	^∗^	0.13		0.06	
Supramarginal	-0.25	+	0.14		-0.05		-0.24	+	0.12		-0.05	
Postcentral	-0.29	^∗^	0.08		-0.11		-0.29	^∗^	0.06		-0.11	
Precuneus	-0.30	^∗^	0.08		0.06		-0.30	^∗^	0.06		0.06	
**Occipital Lobe**												
Lateral Occipital	-0.33	^∗^	-0.05		-0.03		-0.34	^∗^	-0.09		-0.03	
Lingual	-0.34	^∗^	0.17		-0.08		-0.34	^∗^	0.15		-0.08	
Cuneus	-0.26	+	0.02		-0.01		-0.26	+	0.00		-0.01	
Pericalcarine	-0.26	+	0.14		0.00		-0.25	+	0.13		0.00	
**Insula**												
Insula	-0.18		0.14		0.02		-0.17		0.13		0.02	
**Cingulate**												
Rostral Anterior	-0.04		0.07		-0.01		-0.03		0.07		-0.01	
Caudal Anterior	-0.06		0.23	+	0.12		-0.04		0.23	+	0.12	
Posterior	-0.29	^∗^	0.28	^∗^	0.05		-0.28	^∗^	0.27	+	0.06	
Isthmus	-0.22		0.02		0.11		-0.22		0.00		0.11	
**Corpus Callosum (CC)**												
Posterior	-0.35	^∗∗^	0.06		0.10		-0.35	^∗^	0.03		0.11	
Mid-Posterior	-0.59	^∗∗^	0.07		0.01		-0.59	^∗∗^	0.03		0.02	
Central	-0.59	^∗∗^	0.13		0.01		-0.59	^∗∗^	0.10		0.02	
Mid-Anterior	-0.58	^∗∗^	0.10		0.03		-0.57	^∗∗^	0.06		0.04	
Anterior	-0.62	^∗∗^	-0.08		0.13		-0.63	^∗∗^	-0.17		0.17	
**Subcortical**												
Hippocampus	-0.47	^∗∗^	0.23	+	0.00		-0.46	^∗∗^	0.22		0.00	
Amygdala	-0.36	^∗∗^	0.34	^∗^	-0.13		-0.35	^∗^	0.34	^∗^	-0.14	
Caudate	0.21		0.21		-0.08		0.24	+	0.24	+	-0.08	
Putamen	-0.31	^∗^	0.37	^∗∗^	0.21		-0.31	^∗^	0.37	^∗∗^	0.23	+
Pallidum	-0.19		0.08		0.15		-0.19		0.07		0.15	
Accumbens	-0.30	^∗^	0.21		-0.20		-0.29	^∗^	0.20		-0.21	
Thalamus Proper	-0.46	^∗∗^	0.24	+	0.19		-0.46	^∗∗^	0.23	+	0.22	
Brain-Stem	-0.31	^∗^	0.10		0.18		-0.30	^∗^	0.08		0.19	
Ventral Diencephalon	-0.44	^∗∗^	0.17		0.13		-0.44	^∗∗^	0.15		0.15	
**Ventricle**												
Lateral Ventricle	0.69	^∗∗^	-0.20		-0.02		0.69	^∗∗^	-0.19		-0.03	
Inferior Lateral Ventricle	0.63	^∗∗^	-0.05		0.05		0.63	^∗∗^	0.00		0.06	
3rd Ventricle	0.62	^∗∗^	-0.06		0.08		0.62	^∗∗^	-0.01		0.10	
4th Ventricle	0.35	^∗∗^	-0.10		-0.06		0.35	^∗^	-0.07		-0.07	

*N = 54 ^+^*p* < 0.05, ^∗^*p* < 0.025, ^∗∗^*p* < 0.005 (one-tailed).*

The effects of age and fitness on the subcortical ROIs are reported in **Table 2**. The volumes of the hippocampus, amygdala, putamen, and thalamus were associated with both age and fitness. The nucleus accumbens, brain-stem and ventral diencephalon were associated with age but not eCRF. Lastly, age was positively associated with all ventricle measurements, with no corresponding association with fitness.

#### Anatomical Factor Analysis

**FIGURE 2:**
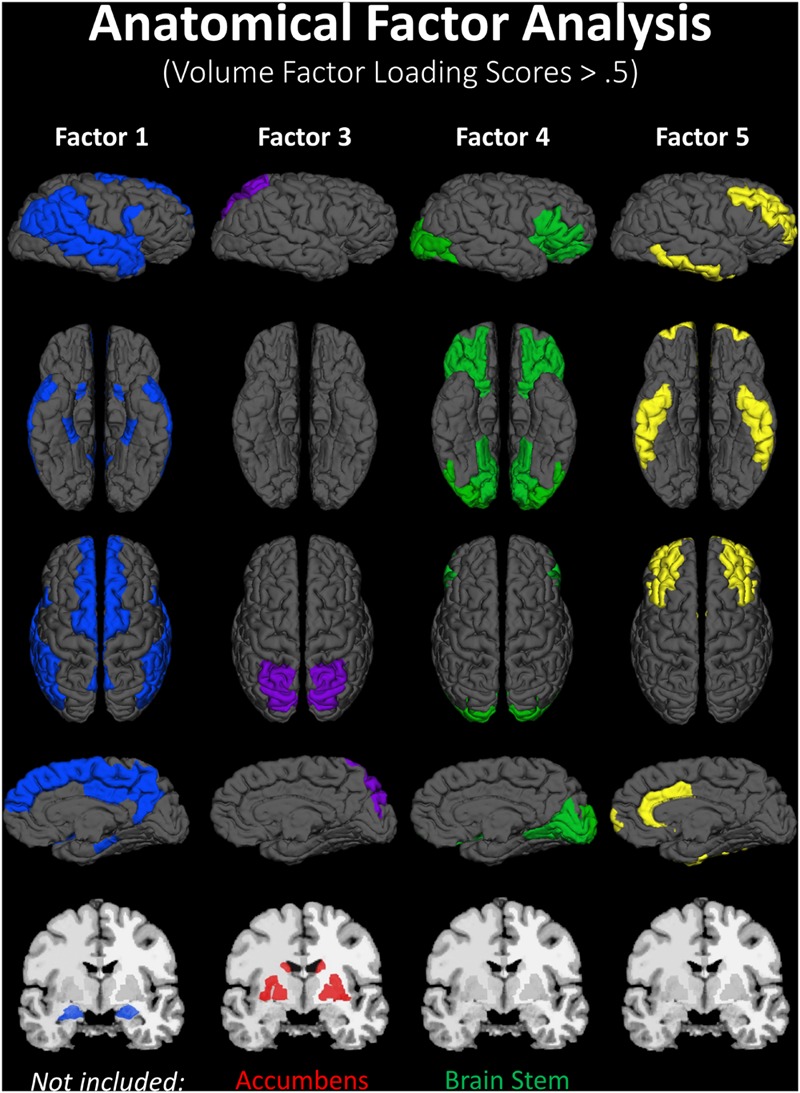
**Visualization of the ROIs loading highly (>0.5) on each factor of the PCA (from data presented in **Table 3**).** Factor 2 (not represented here) is related to ventricles and corpus callosum size (for which loadings were positive and negative, respectively). For factor 3, significant and positive factor loadings were colored in red, with significant but negative factors colored in purple. Superior and inferior views are presented with the anterior portion of the brain pointing upward. Although left and right volumes were not examined separately, for illustration purposes both hemispheres are included in the figure. Anatomical ROIs that had high factor loadings but could not be displayed are listed at the bottom of each factor column.

A lack of overlap between age and CRF effects would be best demonstrated by a double-dissociation, in which some areas show effects of one independent variable and not the other, and some show the opposite effect. However, such a demonstration may be obscured by the large number of comparisons, due to the many areas that are being considered. To better identify which regions tended to covary in volume, and to reduce the number of comparisons in the age and fitness analyses, the volumes of the cortical and subcortical ROIs from the Desikan–Killiany atlas (Jack et al., 1989; Desikan et al., 2006) were submitted to a PCA. Scree plots suggested five components, or factors, which were subjected to Varimax rotation. A visualization of the regions loading on each factor (Factor Loading Score >0.5) is presented in **Figure 2**. Factor loadings of 0.3 or higher after rotation for each of the five components are reported in **Table 3**. The component scores were then submitted to a multiple regression analysis, whose results are presented in **Table 4**, using age, eCRF and the age × CRF interaction term as predictors.

**Table 3:** Factor loadings for anatomical ROIs entered in the factor analysis.

Factor 1		Factor 2		Factor 3		Factor 4		Factor 5	
Supramarginal	**0.73**	CC Mid-Anterior	**0.83**	Putamen	**0.66**	Cuneus	**0.80**	Rostral Ant. C.	**0.67**
Sup. T.	**0.72**	CC Central	**0.80**	Pallidum	**0.62**	Pericalcarine	**0.77**	Rostral Mid. F.	**0.63**
Precuneus	**0.67**	CC Mid-Posterior	**0.79**	Caudate	0.55	Parorbitalis	**0.61**	F. Pole	**0.60**
Mid. T.	0.59	CC Anterior	**0.78**	Accumbens	0.55	Brain Stem	**0.61**	Caudal Ant. C.	0.56
Inferior P.	0.59	CC Posterior	**0.69**	Banks STS	0.32	Lat. Occipital	0.57	Inferior T.	0.54
Banks STS	0.58	Thal. Proper	0.48	Paracentral	0.32	Lingual	0.54	Isthmus C.	0.45
Pars opercularis	0.57	Parahippocampal	0.45	Thal. Proper	0.31	Insula	0.53	Med. Orbito F.	0.40
Paracentral	0.56	Hippocampus	0.44	Precuneus	-0.45	Pars triangularis	0.51	Entorhinal	0.36
Posterior C.	0.56	Precentral	0.36	Isthmus C.	-0.45	Lat. Orbito F.	0.51	Mid. T.	0.34
Insula	0.55	Brain Stem	0.33	Sup. Parietal	-0.55	Med. Orbito F.	0.48	Inferior Parietal	0.31
Parahippocampal	0.54	Postcentral	0.31			Sup. F.	0.46	Fusiform	0.30
Amygdala	0.53	CSF	-0.41			Accumbens	0.43		
Sup. F.	0.53	4th V.	-0.45			Precentral	0.33		
Transverse T.	0.49	Inferior Lat. V.	**-0.70**						
Postcentral	0.47	3rd V.	**-0.73**						
Fusiform	0.46	Lat. V.	**-0.73**						
Putamen	0.44								
Sup. Parietal	0.42								
Thal. Proper	0.41								
Caudal Mid. F.	0.40								
Med. OrbitoF.	0.37								
Caudal Ant. C.	0.37								
Lat. OrbitoF.	0.36								
Hippocampus	0.36								
Precentral	0.34								

*Factors are ranked in descending order. Highlighted cells contain values greater than or less than 0.50. Bolded values are loading scores greater than 0.60. Values less than 0.30 are excluded for ease of presentation. F, Frontal; T, Temporal; C, Cingulate; V., Ventricle; P, Parietal; CC, Corpus Callosum; Thal, Thalamus; Lat, Lateral; Sup, Superior; Mid, Middle; Med, Medial.*

**Table 4:** Raw and partial correlations between anatomical factor loadings with age, eCRF, and eCRF by age interaction (centered age multiplied by centered fitness scores).

	Raw correlation						Partial correlation				
	Age (r)		eCRF (r)		Age × eCRF (r)		Age (β)		eCRF (β)		Age × eCRF (β)
**Anatomical Factors**											
Factor 1:	-0.31	^∗^	0.31	^∗^	0.04		-0.29	^∗^	0.29	^∗^	0.05
Factor 2:	-0.70	^∗∗^	0.08		0.03		-0.70	^∗∗^	0.03		0.05
Factor 3:	-0.01		0.26	+	0.00		0.01		0.26	+	0.00
Factor 4:	-0.24	+	0.04		0.03		-0.24	+	0.02		0.03
Factor 5:	0.03		-0.02		-0.03		0.03		-0.02		-0.03

*N = 54 ^+^*p* < 0.05, ^∗^*p* < 0.025, ^∗∗^*p* < 0.005 (one-tailed).*

The first factor consists of areas that cover most of the medial and lateral cortex. It also contains subcortical regions including the thalamus, putamen and amygdala. Regression analyses revealed that variance in Factor 1 amplitude was associated with both age and eCRF. The second factor (not shown in **Figure 2**) included the CC and ventricles, which were strongly associated with age, but not with eCRF. The third factor was made up almost exclusively of the basal ganglia with an additional contribution by the superior parietal cortex. This factor was positively associated with eCRF, but not age. The fourth factor was comprised of inferior frontal and occipital regions. This factor was associated with age, but was not associated with fitness. The fifth factor comprised regions around the dorsolateral prefrontal cortex, the anterior cingulate, and the inferior temporal lobe. Surprisingly, these regions were not associated with either age or fitness. Thus, we found one factor (Factor 1) that was affected by both aging and fitness, two factors (Factors 2 and 4) that were affected only by aging, one factor (Factor 3) that was affected only by fitness, and one factor (Factor 5) that was not affected by either variable.

### Relationship between Age and Fitness Effects

**FIGURE 3:**
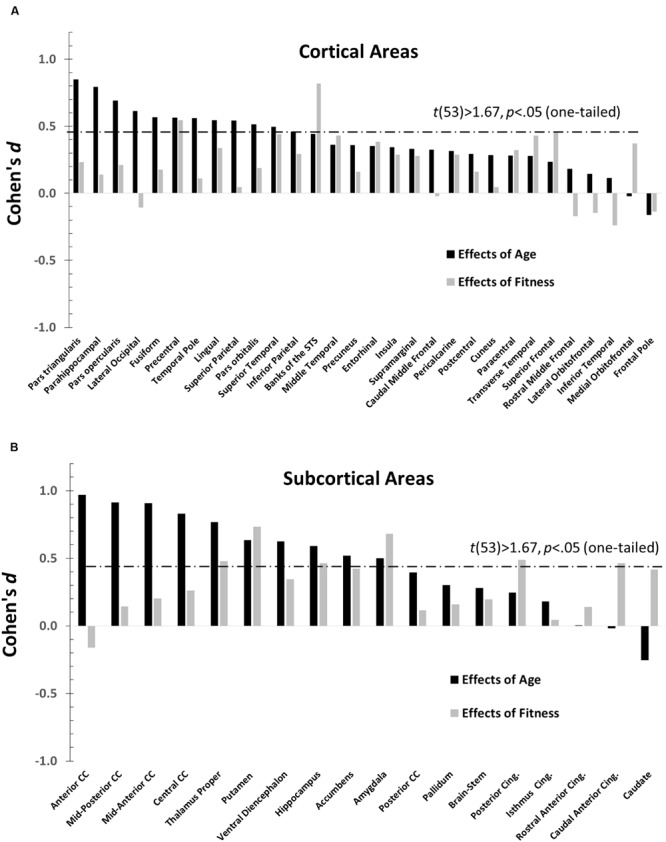
**Bar graphs depicting the Cohen’s *d* (effect sizes) as a function of age (black), and CRF (gray) for all the ROIs examined (defined by the Desikan–Killiany atlas).** For each graph, regions are separately arranged according to decreasing age effects, with regions most impacted by age on the far left. The horizontal dashed line represents the significance threshold for a one-tailed *t*-test, *t*(53) = 1.67, *p* = 0.05. **(A)** Cohen’s *d′*s for normalized cortical regions. **(B)** Cohen’s *d′*s for normalized subcortical gray and white matter regions.

The main purpose of this paper is to evaluate the overlap between the profiles of age and fitness effects across brain regions. The effect sizes (Cohen’s *d*), across different anatomical areas, for high-fit and low-age participants are presented in **Figure 3** (a = cortical and b = subcortical regions). Positive values in this figure indicate age-related reductions in volume (black bars) or fitness-related increases in volume (gray bars), and vice-versa for negative values. Significant effects [*t*(53) > 1.67, *p* < 0.05, one-tailed] are denoted by bars crossing the horizontal dashed line. Nine (out of 48) regions showed significantly greater volumes in high- than in low-fit participants. Twenty-one (out of 48) regions showed significantly greater volumes in the younger than in the older participants.

**FIGURE 4:**
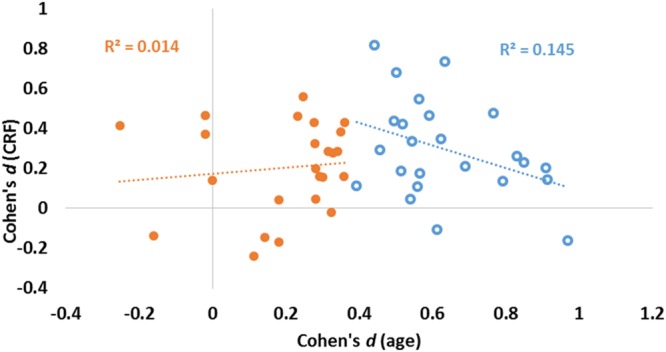
**Scatter plot reporting effect sizes (Cohen’s *d*) for age (abscissa) and CRF (ordinate) for each of the 48 ROIs.** Linear regression lines and coefficients of determination (*R*^2^) computed across regions are also displayed separately for areas with high (blue) and low (orange) aging effect sizes (as determined by a median split of age effect sizes).

The data presented in **Figure 3** suggest that age and fitness effects were especially different in regions that were most impacted by age (left side of **Figures 3A,B**). This was especially true for the CC and ventral frontal regions. To characterize the relationship (or lack thereof) between the two types of effects, a scatter-plot displaying group-level age effects against group-level fitness effects across brain regions is presented in **Figure 4**. Overall, the correlation between these average *t*-scores was not significant, *r*(46) = 0.078, *p* = 0.597.

To quantify the relationship between the two profiles, we computed a consistency-based two-way mixed intra-class correlation coefficient. The result of this analysis indicate that there is little consistency between the profile of age effects and the profile of fitness effects on brain volumes, *r*_ic_= 0.078, *p* = 0.298. Although this analysis failed to reach significance when examining all regions, a median split on the effect sizes of age effects revealed interesting differences. For those regions most significantly impacted by age (blue circles in **Figure 4**), a marginal, but *negative* association was found between age and fitness effects [*N* = 24, *r*(22) = 0.380, *p* = 0.067]. For regions less impacted by age (orange circles in **Figure 4**), no association was found between age and fitness effects [*N* = 24, *r*(22) = 0.117, *p* = 0.586].

## Discussion

The main goal of this study was to determine the overlap between the profiles of the effects of fitness and age on the volumes of different brain areas in adults aged 55 and older. This may have theoretical and practical importance: If age and fitness were found to be related to similar (albeit opposite) volumetric profiles, it could be argued that fitness (or lack thereof) may be an important global mediator of the effects of aging, and a fitness-based intervention could be used to halt, delay or reverse the effects of aging on brain volumes. Although a large number of studies have investigated the effects of aging and those of fitness on the volumes of various brain regions (see Voss et al., 2013 for a detailed review), a determination of the degree of overlap between these two sets of effects is still lacking.

As found previously in several other studies, our results indicate that both age and fitness have effects on brain volumes. As in previous work (Raz, 2005; Raz and Rodrigue, 2006; Raz et al., 2007; Fjell et al., 2013), the effects of age were particularly evident for the ventricles, the CC, some subcortical gray structures such as the hippocampus and portions of the basal ganglia, as well as a number of cortical regions. Also confirming previous work (Gordon et al., 2008; Erickson et al., 2009, 2011; Chaddock et al., 2010; Bugg and Head, 2011; Verstynen et al., 2012), eCRF was associated with larger volumes in a small set of brain structures, including some cortical (precentral gyrus and superior temporal sulcus) and subcortical (amygdala and some portions of the basal ganglia) structures.

Importantly, however, three findings from our study suggest that the profiles of the effects of aging and fitness are not entirely overlapping. First, an intraclass correlation analysis failed to show a significant association between the two types of effects. Second, the multiple regression analysis of the anatomical PCA factor scores showed that age and fitness were by and large correlated with different components. Specifically, although factor 1 was associated with both variables, factors 2 and 4 were associated with age and not with fitness, and factor 3 (comprised mainly of the basal ganglia) was associated with fitness but not age. The results of the PCA, together with the multiple regression analysis, suggest that age has broad effects on much of the cortex. These are, however, at times separable from the effects of fitness, which are more localized to regions such as the basal ganglia and MTL. Third, very few interactions (no more than could be predicted on the basis of chance) were observed between age and fitness, suggesting that fitness did not moderate the age effects. In other words, according to an additive factor logic, this suggests that age and fitness contribute independent, additive effects to brain anatomy.

It is also interesting to note that those regions that are most impacted by age (blue circles on the right of **Figure 4**) are among those not significantly impacted by fitness. As mentioned previously, when considering only the 24 regions showing the largest age effects (top half), a marginally significant negative association between age and fitness effects is revealed. Although it is not entirely clear why this happens, it is possible that, when the effects of age become too prominent, then the effects of fitness can no longer counteract them. An alternative explanation is that, in some cases, area volume may not be the best measure of tissue preservation, and that other indices of anatomical reserve (such as cortical thinning or measures of myelination) may be more sensitive. This may account for the lack of correlation between CC volume and fitness obtained in our sample, while an association between fitness and white matter integrity (as measured by fractional anisotropy) has been reported in previous studies (e.g., Johnson et al., 2012; Chaddock-Heyman et al., 2014). It should be noted, however, that neither of these studies directly compared the effects of age and fitness on white matter integrity. In addition, the sensitivity explanation does not account for the fact that we still find a strong relationship between age and white matter preservation in our study.

There is of course ample evidence of both physiological (Burdette et al., 2010; Fabiani et al., 2014; Zimmerman et al., 2014) and cognitive (Colcombe and Kramer, 2003; Kashihara et al., 2009; Erickson et al., 2011; Weinstein et al., 2012) benefits of physical fitness in older adults. Further, the brain structures that we (as well as others) find to be associated with CRF actually support important functional phenomena (Gordon et al., 2008; Erickson et al., 2009, 2010; Chaddock et al., 2010; Bugg and Head, 2011; Verstynen et al., 2012; Weinstein et al., 2012).

A number of mechanisms might help explain why some regions seem to be more “fitness sensitive” compared to others. First, many of these “fitness sensitive” regions play unique roles in the planning, coordination, and execution of movements required during aerobic exercise. Indeed, many of the fitness-sensitive regions found in this study are related to motor functions including the precentral gyrus (primary motor cortex), basal ganglia (regulation of movement and motor control), superior temporal sulcus (perception of biological motion), and anterior cingulate (coordination of motor behavior) (DeLong et al., 1984; Grossman and Blake, 2001; Wenderoth et al., 2005; Colcombe et al., 2006; Gordon et al., 2008; Chaddock et al., 2010; Verstynen et al., 2012). Thus, it is possible that for those older adults who exercise, increased use of these regions might preserve them from age-related atrophy, contributing to regional variations.

A second possible contributor to this regional specificity is the fact that neurogenesis is restricted to just a few regions within the brain. Animal studies have shown that CRF induces neurogenesis in rodents, although this phenomenon seems to be restricted to the hippocampus (e.g., Altman, 1969; Brown et al., 2003; Farmer et al., 2004; van Praag, 2008). In humans adult neurogenesis has also been shown within the hippocampus and regions of the basal ganglia (Eriksson et al., 1998; Spalding et al., 2013; Ernst et al., 2014). In the current study and a number of prior investigations, the effects of fitness on the hippocampus and the basal ganglia are particularly pronounced (e.g., Erickson et al., 2009; Chaddock et al., 2010; Ahlskog, 2011; Bugg and Head, 2011; Erickson et al., 2011; McAuley et al., 2011; Nagamatsu et al., 2016). In fact, our study showed that regions of the basal ganglia (particularly the putamen) demonstrated the strongest association with eCRF, after partialing out the effects of age and gender.

Several other mechanisms could further explain the variations in fitness sensitivity of certain regions including: differences in cerebral blood flow and arterial stiffness across the cortex (e.g., Fabiani et al., 2014; Zimmerman et al., 2014); variations in the sensitivity to various neurotrophic factors (e.g., Voss et al., 2013); and differential oxygen requirements across regions (Kreisman et al., 2000). Indeed, it is likely that these mechanisms might work synergistically to prevent atrophy in some regions, while providing minimum influence on other areas. Our study, however, points out that fitness cannot completely offset the declines associated with normal aging, and illustrates a possible approach for future studies to examine the interactions between fitness and age on volumes across different brain structures.

Within this context, it is important to note that our study, by and large, replicates most of the major findings that have been reported both in the literature on anatomical brain aging as well as in the effects of fitness on brain volumes. This indicates that the results of our study are not outliers with respect to the extant literature. What is novel here is the profile approach used, and the consequent types of analyses that were carried out to examine it. A critical requirement for these analyses is the inclusion of a large number of areas distributed across the whole brain. Another feature of the current study is the dichotomization of the most critical independent variables (age, fitness, and gender), which permits their orthogonalization. This allows for a comprehensive and independent examination of the profiles of the effects of these variables (and of their interactions) across areas. Finally, another important feature of the analytic strategy employed in this study is the use of a factor-analysis approach to identify the brain structures that covary as a function of age and fitness. Although this is a data-driven (rather than theoretically based) approach, and therefore it is inherently exploratory, it does help reduce multiple comparisons. It could also help guide future research aimed at understanding the factors underlying the emergent grouping (as well as the stability of the grouping across different samples).

A possible limitation of the current work is the relatively small sample size (*N* = 54), which could diminish the power of the analysis. Since some of the findings depend on tests of the null hypothesis (such as the lack of a significant intra-class correlation), this lack of power may potentially be an important problem. However, it should be noted that the statistical power of a study depends not only on sample size but also on the extent to which experimental error can be controlled or minimized. In this study we used a very rigorous process of data quality control, as well as methods for orthogonalizing the variance due to age and CRF. The effectiveness of these procedures is demonstrated by our replication of the main significant effects for both age and fitness normally reported in the literature.

## Conclusion

The current study presents an analytic approach to investigate the degree of overlap of the effects of critical variables on brain anatomy. A crucial aspect of this work is the orthogonalization of the effects of these two variables. This allows for the separate study of the profiles of the effects associated with age and fitness, and demonstrates that they are only partially overlapping. While some areas (such as the precentral gyrus, the banks of the superior temporal sulcus, and some subdivisions of the MTL) are affected by both age and fitness, there are a number of areas (including extensive regions of the frontal, parietal, and temporal cortex, as well as the CC) that are only affected by aging, and some structures (mostly in the basal ganglia) that are uniquely affected by fitness.

These findings support the idea that aging and fitness (or lack thereof) have differential effects on the brain. Understanding that fitness cannot revert all of the effects of aging is important for many reasons. First, it may lend support to the hypothesis that a comprehensive preventive approach to brain aging focusing on other lifestyle factors (such as diet or cognitive training) in addition to fitness may be more effective than fitness interventions alone, as these other factors may potentially help protect those brain regions not responsive to fitness. Second, the limited overlap of these effects in many regions may help explain the differential effects of fitness on the various domains of cognition found in previous studies. Finally, these differences may also help guide neurobiological research examining, in an area-specific way, the mechanisms by which fitness could impact the brain.

## Author Contributions

MF and GG designed the study and directed all aspects of the project, and helped write and edit the manuscript. MAF with the help of RB conducted all anatomical analyses, and MAF wrote the first version of this manuscript. KAL, BZ, HT, and NS-G carried out the data collection. AG and BPS provided consulting on FreeSurfer. Everyone commented on the paper.

## Conflict of Interest Statement

The authors declare that the research was conducted in the absence of any commercial or financial relationships that could be construed as a potential conflict of interest.

## Footnotes

1 http://surfer.nmr.mgh.harvard.edu/fswiki/FsTutorial/TroubleshootingData

## Abbreviations

CRF: cardiorespiratory fitness
eCRF: estimated cardiorespiratory fitness
eTIV: estimated total intracranial volume
VO_2max_: maximal oxygen uptake
